# The dance of macrophage death: the interplay between the inevitable and the microenvironment

**DOI:** 10.3389/fimmu.2024.1330461

**Published:** 2024-03-20

**Authors:** Magdalena Makuch, Mariia Stepanechko, Małgorzata Bzowska

**Affiliations:** Department of Immunology, Faculty of Biochemistry, Biophysics, and Biotechnology, Jagiellonian University, Kraków, Poland

**Keywords:** macrophages, cell death, pyroptosis, necroptosis, ferroptosis, tumor-associated macrophages, atherosclerosis, cancer

## Abstract

Macrophages are highly plastic cells ubiquitous in various tissues, where they perform diverse functions. They participate in the response to pathogen invasion and inflammation resolution following the immune response, as well as the maintenance of homeostasis and proper tissue functions. Macrophages are generally considered long-lived cells with relatively strong resistance to numerous cytotoxic factors. On the other hand, their death seems to be one of the principal mechanisms by which macrophages perform their physiological functions or can contribute to the development of certain diseases. In this review, we scrutinize three distinct pro-inflammatory programmed cell death pathways – pyroptosis, necroptosis, and ferroptosis – occurring in macrophages under specific circumstances, and explain how these cells appear to undergo dynamic yet not always final changes before ultimately dying. We achieve that by examining the interconnectivity of these cell death types, which in macrophages seem to create a coordinated and flexible system responding to the microenvironment. Finally, we discuss the complexity and consequences of pyroptotic, necroptotic, and ferroptotic pathway induction in macrophages under two pathological conditions – atherosclerosis and cancer. We summarize damage-associated molecular patterns (DAMPs) along with other microenvironmental factors, macrophage polarization states, associated mechanisms as well as general outcomes, as such a comprehensive look at these correlations may point out the proper methodologies and potential therapeutic approaches.

## 1 Introduction

Macrophages, ever since Elie Metchnikoff’s pioneering discovery of their phagocytic capabilities in the late 19th century, have occupied a central role in immunology. Present in nearly every tissue, these versatile professional phagocytes perform multifaceted functions in antimicrobial defense, shaping the immune response through antigen presentation and cytokine secretion. However, it is worth noting that macrophages’ roles extend well beyond direct participation in antimicrobial defense, and over the past three decades research has unveiled a multitude of fascinating functions of these cells. We are now gaining a deeper understanding of the diverse and highly specialized roles that macrophages play as pivotal contributors to the clearance of senescent or dead cells, tissue repair, regeneration, and the maintenance of tissue integrity and homeostasis (1).

One of the distinctive characteristics of macrophages is their remarkable plasticity and adaptability, allowing them to fulfill the unique demands of the surrounding tissue microenvironment. The tissue-specific diversity of macrophages derives from phenomena such as their embryonic origin or recruitment from circulating monocyte pools, and prior encounters with conditions like inflammation or injury. All these experiences leave a lasting imprint on cell identity (2–4). Consequently, this led to the development of macrophage classification, which is based on their possible mechanisms of activation and subsequent roles in either promoting or resolving inflammation. Macrophage polarization states were divided into M1 and M2 phenotypes, commonly referred to as classically and alternatively activated. The M1 type is induced by LPS and IFNγ, and associated with enhanced phagocytic activity, antigen presentation, increased reactive oxygen species (ROS) production, and the release of pro-inflammatory cytokines. Conversely, following the downstream signals of cytokines such as IL-4 and IL-13 macrophages polarize into the M2 phenotype, which results in an anti-inflammatory response that promotes angiogenesis, tissue remodeling, and repair (5). Such a view, however, is regarded now as too dichotomic and rather applicable to polarized murine macrophages, which are easier to distinguish (6). In contrast, the macrophage polarization scheme derived from human studies seems to be much more complex, dependent on various contexts (7), and reversible (8). A good illustration is the possibility of differentiating M2a, M2b, M2c, and M2d subsets. They occur upon induction with various stimuli, yet all are characterized by the ability to secrete high levels of anti-inflammatory IL-10 (7, 9). Indeed, M2a type is induced by IL-4/IL-13, whereas M2b by immune complexes and LPS/IL-1β. On the other hand, IL-10/TGFβ and glucocorticoids polarize macrophages toward M2c, while TLR+adenosine A_2_ receptor agonists induce M2d phenotype (7, 9). The challenges linked to the classification of macrophage phenotypes have been extensively discussed (5, 10). We highlight that M1- and M2-like macrophages are not mutually exclusive and frequently coexist. Moreover, the diverse nature of stimuli, the interaction of various pathways, and the lack of specific markers suggest that macrophages may form mixed phenotypes, rather than stable subsets (5). Later in the text, we describe the role of macrophage cell death types in their polarization as well as the progression of two human diseases – atherosclerosis and cancer. We decided to use the simplified phenotype classification according to that employed by the authors. Nevertheless, we emphasize that this represents a form of simplification.

Macrophage states are the results of various phenomena that cells encounter throughout their life. All of them profoundly influence macrophage physiology and can finally culminate in cell death. Noteworthy, there are various, other than apoptosis, forms of programmed cell death (PCD), which are controlled by multiple discrete yet interconnected signaling pathways (11, 12). In the first part of this review, we describe the mechanisms of three different pro-inflammatory PCD modalities – pyroptosis, necroptosis, and ferroptosis – with a particular emphasis on their occurrence in macrophages under specific circumstances. All these cell deaths have profound implications not only for the affected cell but, more importantly, for the microenvironment, tissue homeostasis, prolongation of inflammation, and progression of diseases. In the second part of our review, we meticulously examine two such maladies – atherosclerosis and cancer. Our attention is directed toward them, as there is a considerable amount of data showing a significant impact of both macrophage death and inflammation in their pathogenesis and treatment. Moreover, evidence from both diseases implied a connection between the occurrence of specific inflammatory PCD and macrophage polarization state. For the first time in one place we gather information on specific triggering factors, microenvironmental conditions, pathways, and their crosstalk, which result in the dance of macrophages with death. Understanding the mechanisms underlying macrophage pyroptosis, necroptosis, and ferroptosis is of paramount significance since they may constitute attractive targets for potential therapies.

## 2 Pyroptosis, necroptosis, and ferroptosis: mechanisms in macrophages

Pyroptosis, necroptosis, and ferroptosis represent distinctive forms of programmed cell death differing from silent apoptosis, as they are recognized for their pro-inflammatory and immunogenic roles. Morphologically, both pyroptosis and necroptosis involve cell swelling, although the first one generally results in milder cellular content leakage compared to the second one. In both cases, however, cell membrane damage is induced by oligomerization of pore-forming effector protein – GSDMD in pyroptosis and MLKL in necroptosis. It signifies a similar effector mechanism leading to the release of intracellular material, such as pro-inflammatory cytokines IL-1β and IL-18 and/or damage-associated molecular patterns (DAMPs) (13). By contrast, little information is available regarding the integrity of cell membrane in ferroptosis. It is thought, however, that at the late stage of this cell death plasma membrane rupture may occur after prior cell swelling (14). Nevertheless, both the progression and execution of ferroptosis are underpinned by disturbances in bioenergetic metabolism as well as the disruption of redox homeostasis, which closely resembles the mechanisms observed in necroptosis (15). Another common feature of these two PCDs is that macrophages seem to exhibit varying susceptibilities to undergo them, depending on the cell polarization state. Further elaboration on this aspect is provided at the end of this section.

### 2.1 Pyroptosis

The name ‘pyroptosis’ derives from Greek, where *pyro* refers to fire or fever, and *ptosis* means failing. It was first described in 1986 by Friedlander, who observed that primary mouse macrophages treated with anthrax lethal toxin (LT) underwent cell death accompanied by the release of cellular content (16). Later, pyroptosis was described by Zychlinksy et al., who indicated that *Shigella flexneri* infection results in suicide in macrophages, however, this cell death was initially referred to as apoptosis (17). These findings were further examined by Chen et al., who in 1996 reported that *S. flexneri*-infected macrophages were characterized by activation of interleukin-1β-converting enzyme (ICE, caspase-1) (18). ICE was discovered for the first time in 1989 (19) and subsequently associated with the ability to process interleukin-1β precursor into its mature form (20, 21). Such a significant role of ICE in the described form of programmed cell death led Cookson et al. to propose the term pyroptosis (22).

**Figure 1:**
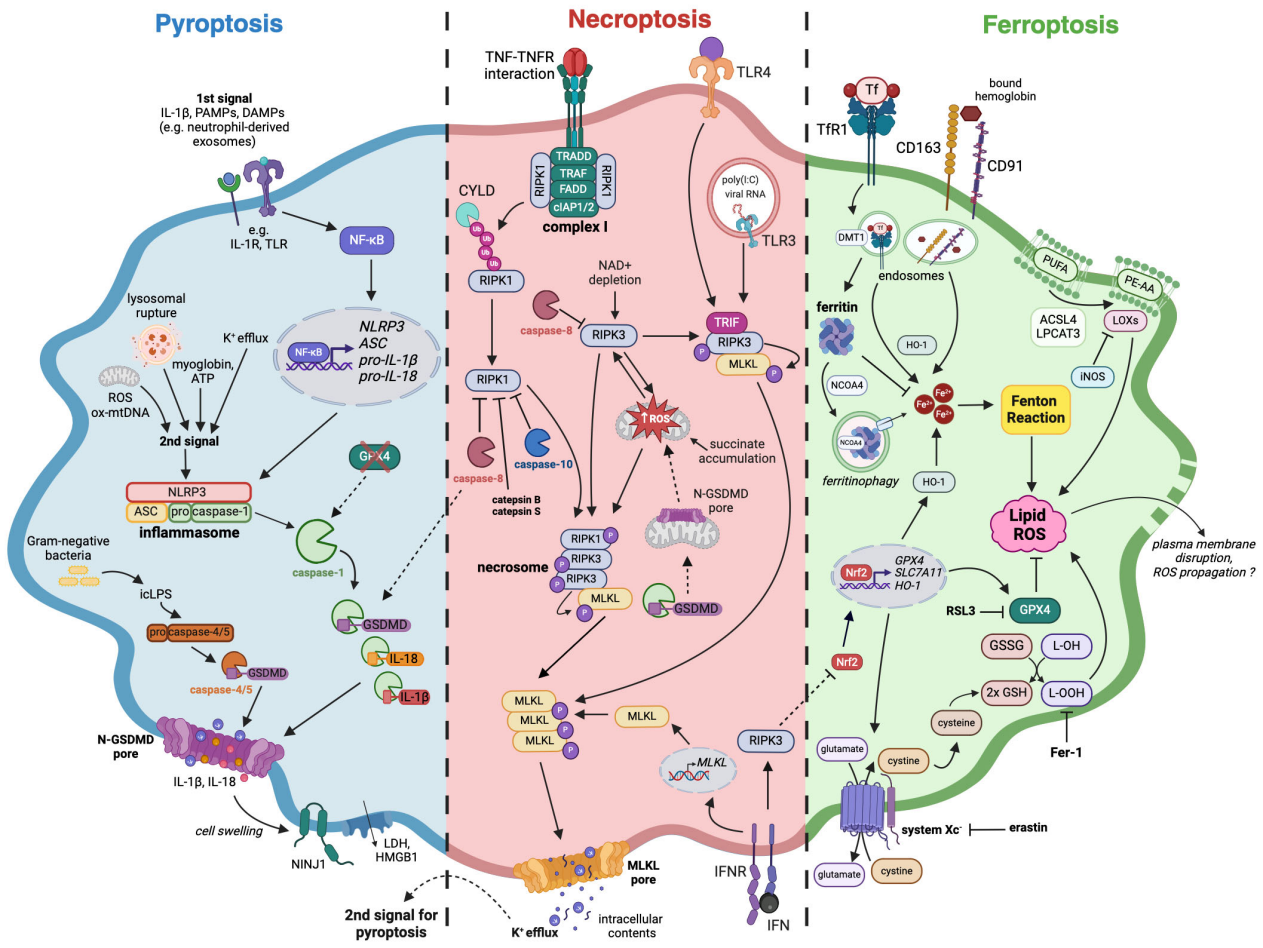
The mechanisms of pyroptosis, necroptosis, and ferroptosis in macrophages. A large number of studies confirmed that macrophages are capable of following different immunogenic programmed cell death pathways upon various stimuli. Pyroptosis (blue) induction requires two signals, which eventually lead to the assembly of inflammasome complex and activation of caspase-1, followed by IL-18, IL-1β as well as GSDMD cleavage and maturation. Necroptosis (red) can arise after TNF or TLR stimulation as a switch from apoptosis when there is a simultaneous caspases inhibition. Necrosome is created as a result of RIPK1 and RIPK3 autoactivation or direct interaction of TRIF adaptor protein with RIPK3, followed by phosphorylation of the effector protein, which is MLKL. Both pyroptosis and necroptosis, despite requiring different stimuli for their activation, are characterized by the presence of membrane pores, formed by N-GSDMD and pMLKL respectively. Additionally, cell swelling-associated events that happen in pyroptosis can activate NINJ1, leading to plasma membrane rupture. The subsequent release of cellular contents like HMGB1 further stimulates immune response. Ferroptosis (green) occurs as an outcome of iron homeostasis disruption and an increase in the labile iron pool that can undergo Fenton reaction, leading to lipid peroxidation and subsequent cell death. An iron-storing protein ferritin binds free iron, thereby impeding it from entering the reaction. Amino acid antitransporter system Xc- and GPX4 can alleviate oxidative stress and prevent ferroptosis while their blockade by erastin and RSL3 respectively diminishes cellular antioxidative defense. The loss of cell membrane integrity during ferroptosis has not been well documented, however, it is thought that plasma membrane disruption may occur. Dashed lines indicate crosstalk between pyroptotic, necroptotic, and ferroptotic signaling pathways. Pyroptosis: ASC – apoptosis-associated speck-like protein containing a CARD, DAMPs – danger-associated molecular patterns, GSDMD – gasdermin D, HMGB1 – High mobility group protein B1, icLPS – intracellular lipopolysaccharides, IL-1β – interleukin-1β, IL-18 – interleukin-18, IL-1R – interleukin-1 receptor, LDH – lactate dehydrogenase, NF-κB – Nuclear factor kappa-light-chain-enhancer of activated B cells, NINJ1 – Ninjurin 1, N-GSDMD – GSDMD N-terminal domain, NLRP3 – NLR Family Pyrin Domain Containing 3, ox-mtDNA – oxidized mitochondrial DNA, PAMPs – pathogen-associated molecular patterns, pro-IL-1β – pro-interleukin-1β, pro-IL-18 – pro-interleukin-18, ROS – reactive oxygen species, TLR – Toll-like receptor; Necroptosis: cIAP1/2 – cellular inhibitor of apoptosis protein 1/2, CYLD – cylindromatosis protein, FADD – Fas-associated death domain, IFN – interferon, IFNR – interferon receptor, MLKL – mixed lineage kinase domain-like protein, polyIC – Polyinosinic-polycytidylic acid, RIPK1 – receptor-interacting protein kinase 1, RIPK3 – receptor-interacting protein kinase 3, TLR3 – Toll-like receptor 3, TLR4 – Toll-like receptor 4, TRADD – TNFR-associated death domain, TRAF – TNFR-associated factor, TRIF – TIR-domain-containing adapter-inducing interferon β; Ferroptosis: ACSL4 – acyl-CoA synthase long chain family member 4, DMT-1 – divalent metal transporter 1, Fer-1 – ferrostatin-1, GSH – glutathione, GSSG – glutathione disulfide, GPX4 – glutathione peroxidase 4, HO-1 – heme oxygenase 1, iNOS – inducible nitric oxide synthase, L-OH – lipid alcohol, L-OOH – lipid peroxide, LOXs – lipoxygenases, LPCAT3 – lysophosphatidylcholine acyltransferase 3, Nrf2 – nuclear factor erythroid 2-related factor 2, NCOA4 – nuclear receptor coactivator 4, PE-AA – Phosphatidylethanolamine (PE)-linked arachidonic acid, PUFA – polyunsaturated fatty acids, RSL3 – RAS-selective lethal 3, SLC7A11 – solute carrier family 7 member 11, TfR1 – transferrin receptor 1.

The characteristics of pyroptosis include the activation of inflammasomes and their sensors (also known as pattern recognition receptors, PRRs) as well as the loss of plasma membrane integrity (23). A canonical inflammasome is a multimolecular complex assembled in the cytoplasm after PRRs activation, which can recognize pathogen-associated molecular patterns (PAMPs) or danger-associated molecular patterns (24). PRRs include Nod-like receptor (NLR) family, the DNA receptor Absent in Melanoma 2 (AIM2), and the Pyrin receptor (23). NLRP3 and AIM2 inflammasomes require two signals for their activation (**Figure 1**). The first signal (also known as priming signal) is usually obtained through PAMPs detection by Toll-like receptors (TLR) or induction of cytokine receptors (e.g., IL-1R), and results in NF-κB activation and upregulated transcription of NLRP3, adapter apoptosis-associated speck-like protein (ASC), pro-IL-1β, and pro-IL-18 (25). Interestingly, it has recently been shown that exosomes released from TNF-α-stimulated neutrophils can also act as a priming signal for macrophages (26). The second signal (also known as activation signal) can be triggered by a variety of stimuli including ions efflux/influx, oxidized mitochondrial DNA, lysosomal rupture, reactive oxygen species (ROS), ATP (27), and myoglobin (28). After activation, inflammasome sensors oligomerize and subsequently bind ASC, which contains a caspase activation recruitment domain (CARD) necessary to recruit pro-caspase-1 and assemble inflammasome (23, 24). Such canonical inflammasomes constitute a platform for the autoproteolysis of caspase-1, which activated cleaves pro-IL-1β and pro-IL-18 to their mature forms (**Figure 1**).

Furthermore, caspase-1 cleaves gasdermin D to form N-terminal GSDMD (N-GSDMD) able to oligomerize and perforate cell membrane by creating a transmembrane pore. It subsequently leads to cell swelling, lysis, and pyroptosis accompanied by the release of cellular contents that strongly activate inflammatory responses (24). Gasdermin D pore diameter is about ~21 nm (29), which makes it quite selective about large molecules, but non-selective about smaller cellular contents. However, recent findings indicated the importance of charge displayed by the cargo transported through GSDMD pore. In their study, Xia et al. demonstrated that gasdermin D pore seems to favor the passage of mature IL-1β and IL-18 rather than their proforms. Such phenomenon originates from the fact that IL-1β precursor contains an acidic domain, which is removed by active caspase-1, enabling mature interleukin to cross the negatively charged conduit of the GSDMD pore (30). Moreover, Kayagaki et al. demonstrated that cell swelling, which occurs in macrophage pyroptosis and necrosis, can lead to cell-surface Ninjurin 1 (NINJ1) protein activation (31). Consequently, there is an induction of plasma membrane rupture, followed by the release of lactate dehydrogenase (LDH) and DAMPs such as high mobility group protein B1 (HMGB1), which further intensify inflammation (31).

Pyroptosis can also derive from the non-canonical pathway, which can be activated by intracellular LPS (icLPS) recognized by caspase-11 (in mice) or caspase-4/5 (in humans) by their CARD domain (24). The interaction between icLPS and caspase-4/5/11 results in autoproteolytic activation of caspases, which also cleave GSDMD (**Figure 1**). Subsequently, N-GSDMD oligomerizes and forms membrane pores, leading to K^+^ efflux (23). Interestingly, such ion fluctuation is the signal for NLRP3 inflammasome assembling and caspase-1 activation, which eventually lead to pyroptosis and the release of IL-1β and IL-18. Although caspases-4/5/11 are not capable of cleaving pro-IL-1β and pro-IL-18, they mediate their maturation processing through NLRP3/caspase-1 pathway in some cell types (24).

A growing body of evidence suggests that certain cells with activated inflammasomes and undergoing cell death sometimes do not experience cell lysis. For example, DiPeso et al. showed that pyroptotic cell death in bone marrow-derived macrophages (BMDMs) is not always accompanied by cell rupture, therefore lysis prevention does not couple with inhibition of cell death after a pyroptotic stimulus (32). These data suggest that pyroptosis does not necessarily result in cell lysis, though cell swelling and leaking are still important consequences of GSDMD pores formation (32). Moreover, inflammasome activation and pyroptosis induction in macrophages seem to be reversible. The latest findings demonstrated that endosomal sorting complexes required for transport III (ESCRT III) machinery in macrophages was able to dampen GSDMD pore-mediated cell death and IL-1β release, even though inflammasomes were activated in both canonical and non-canonical ways (33). The authors suggest a potential role of ESCRTs in removing GSDMD pores from the macrophage membrane, thereby indicating that pyroptosis in these cells can be restricted (33).

### 2.2 Necroptosis

The elucidation of necroptosis arose from the initial observation that TNF, a cytokine traditionally associated with the ability to induce apoptosis (34), could also trigger necrotic cell death when various caspases were concurrently inhibited (35). Subsequently, Holler et al. proved that such necrotic cell death could also be initiated by Fas as well as TNF-related apoptosis-inducing ligand (TRAIL), and that active receptor-interacting protein kinase 1 (RIPK1) seemed to be vital for this process (36, 37) This RIPK1-dependent necrotic cell death was termed necroptosis (36), and, despite morphological similarities, was distinguished from necrosis, which is an accidental cell death resulting from irreversible cellular injury. Over time researchers identified other significant molecules downstream RIPK1, including receptor-interacting protein kinase 3 (RIPK3) (38–40) and mixed lineage kinase domain-like protein (MLKL), which serves as the ultimate executor of necroptosis (41).

To describe the exact mechanism of necroptosis, it is inevitable to mention first the TNF-mediated activation and apoptosis pathways. The interaction between TNF and tumor necrosis factor receptor (TNFR) leads to the formation of complex I (**Figure 1**), which is situated at the cell membrane and consists of RIPK1, Fas-associated death domain (FADD), TNFR-associated death domain (TRADD), TNFR-associated factors (TRAFs) as well as cellular inhibitor of apoptosis protein 1 (cIAP1) and 2 (cIAP2). Within complex I, RIPK1 undergoes extensive ubiquitination by TRAFs, cIAPs, and linear ubiquitin chain assembly complex (LUBAC), inhibiting its kinase activity and promoting nuclear factor kappa B (NFκB)-mediated cell survival (42). However, a reduction in RIPK1 ubiquitination, either by deubiquitinating enzyme cylindromatosis protein (CYLD) or inhibition of cIAPs with SMAC mimetic (SM), leads to RIPK1 dissociation from complex I. Subsequently, RIPK1 binds to RIPK3, FADD, TRADD, and caspase-8, thereby forming complex II. Active caspase-8 cleaves downstream caspase-3/7 and RIPK1, inducing cell apoptosis while simultaneously inhibiting necroptosis (43). In the absence of caspase-8 activity, RIPK1 and RIPK3 initiate necroptosis – an alternative, caspase-independent pathway of PCD (**Figure 1**). RIPK1 and RIPK3 interact by their RIP homotypic interaction motifs (RHIMs), undergo autophosphorylation, and form a complex called necrosome. It recruits and phosphorylates MLKL, which oligomerizes and forms pores in the cell membrane, leading to the release of intracellular contents and eventual cell death (41, 44).

Induction of necroptosis by TNF and pan-caspase inhibitor is observed in primary human monocyte-derived macrophages (hMDMs), the U937 monocytic cell line, primary murine peritoneal macrophages (PMs), BMDMs, and the J774A.1 cell line. Interestingly, human THP-1 and murine RAW264.7 cell lines exhibit resistance to described canonical necroptosis stimulation (45, 46) and require additional degradation of cIAPs.

Caspase-8 serves as a pivotal regulator of cell fate, with options spanning across survival, apoptosis, or necroptosis (23) — a role that has been firmly established, also in macrophages (47, 48). The current thinking is that caspase-8 homodimers promote cell death, whereas cleavage events mediated by heterodimers of caspase-8 and cFLIP dictate life (49). Moreover, activation of the death receptor Fas on murine macrophages induces caspase-8-dependent processing of caspase-3 and apoptosis, whereas stimulation of TNFR1, TLR3, or TLR4 does not. However, in the absence of caspase-8, either TNF or TLR3 and TLR4 agonists are lethal to murine macrophages, which undergo cell death mediated by RIPK3 and MLKL (50). Active caspase-8 appears to suppress necroptosis by cleaving RIPK1 and limiting its interaction with RIPK3 (47, 51). Recently, it was also demonstrated that caspase-10 cleaved RIPK1 more efficiently than caspase-8, thus U937 cells lacking caspase-10 were more sensitive to death-inducing stimuli (52). Cathepsins B and S, common proteolytic enzymes in myeloid cells, are also able to cleave RIPK1, thereby efficiently mitigating TNF- or LPS-induced necroptosis in BMDMs (53).

When caspase activity is suppressed, necroptosis can also be triggered upon TLRs stimulation, commonly in human and murine macrophages (**Figure 1**). For TLR2, TLR5, and TLR9 this process depends on TNF release and the autocrine or paracrine activation of TNFR1. In contrast, TLR3 and TLR4 stand out as they rely on the TRIF adaptor protein (TIR-domain-containing adapter-inducing interferon β) in their signaling cascade. Upon exposure to poly-I:C (a ligand for TLR3) or LPS (a ligand for TLR4), the TRIF protein directly interacts with RIPK3 through its RHIM domain, leading to the necrosome formation and the activation of MLKL (54–57). Contrarily to endothelial cells and fibroblasts, RIPK1 in macrophages is implicated in the mechanism of necroptosis induced by TLR3 and TLR4 (54, 55). Interestingly, RIPK1’s ubiquitination pattern, which modulates its interactions with proteins like TRADD, FADD, and TNFR1, exerts dual effects by promoting TNF- but simultaneously restraining TLR-induced necroptosis (58). Furthermore, c-Jun N-terminal kinases (JNKs) serve as additional regulators that facilitate necroptosis in response to TNF and TLR ligands. Strikingly, regardless of their kinase activity, JNKs’ scaffolding function limits TRIF oligomerization and hinders TLR-induced necroptosis (59).

The oligomerization and attachment of phosphorylated MLKL to the cell membrane during necroptosis is not equivalent of cell death. Indeed, similarly to pyroptosis, necroptosis-induced membrane disruptions can be repaired by the ESCRT III machinery, which was confirmed in immortalized macrophages and BMDMs (60, 61). This repair process is more effective in bone marrow-derived dendritic cells than in BMDMs, correlating with their different sensitivities to LPS- and caspase inhibitor-induced necroptosis (61). Surprisingly, once pMLKL translocates to the membrane, phosphatidylserine is externalized, a feature traditionally linked to apoptosis. This precedes the release of vesicles called necroptotic bodies (62). Noteworthy, MLKL protein, via its interaction with ESCRT III machinery, also facilitates endosomal vesicle trafficking independently of necroptotic stimuli (61) as well as plays a role in lipid handling (63) and phagolysosome fusion in macrophages (64).

Reactive oxygen species are firmly implicated in necroptosis across diverse cell types, including macrophages (45, 65–72). Accumulating evidence indicates that ROS act by oxidizing cysteine residues within RIPK1 and MLKL, consequently promoting protein aggregation and necrosome assembly (54, 68). Additionally, activated RIPK3 amplifies the generation of ROS (**Figure 1**), thereby establishing a positive feedback loop that favors necroptosis (67). TNF elicits RIPK3-dependent mitochondrial ROS generation, partially through the activation of the pyruvate dehydrogenase complex (PDC), which regulates oxidative respiration (66). Moreover, excessive TNF triggers robust glutaminolysis, succinate accumulation, and subsequent generation of mtROS via reverse electron transport (RET) at mitochondrial complex I (73). RET, along with NAD+ depletion, plays confirmed roles in TNF-, SMAC mimetic-, and caspase inhibitor-induced necroptosis in BMDMs (69, 74). The triggering receptor expressed on myeloid cells 1 (TREM-1) activation through mTOR-mediated signaling promotes Drp1-dependent mitochondrial fission, mitophagy, and necroptosis of alveolar macrophages during acute lung injury (75). Importantly, elevated TREM-1 expression on myeloid cells has been reported in certain tumors and correlated with worse prognosis (76).

### 2.3 Ferroptosis

Ferroptosis was first described in 2012 by Dixon et al. as iron-dependent cell death that could be triggered by small molecule erastin and inhibited by ferrostatin-1 (Fer-1) (77). Generally, this cell death type is associated with the accumulation of free iron (Fe^2+^), lipid peroxidation (LPO), ROS production, and changes in mitochondrial morphology. Furthermore, ferroptosis can be regulated by amino acid antitransporter system Xc^-^, glutathione peroxidase 4 (GPX4), or nuclear factor erythroid 2–related factor 2 (Nrf2) (78). It is believed that ferroptosis, contrary to earlier described pyroptosis and necroptosis, does not result in plasma membrane rupture, leaving it intact (79). Nevertheless, recent findings suggest that cell membrane rupture indeed takes place and is preceded by cell swelling (14, 80) as well as an increase in cytosolic level of Ca^2+^ (80). Interestingly, Riegman et al. showed that ferroptosis could propagate *in vitro* through the spreading of cell swelling in a lipid peroxide- and iron-dependent manner (14), thus characterizing additional unique features of this cell death pathway. Extensive reviews on either the mechanism of ferroptosis or its differences from other types of regulated cell death may be found elsewhere (78, 81, 82).

As for macrophages, their ferroptosis was first described in murine models of hemochromatosis (83) and transfusion (84). The first study revealed that ferric citrate overload led to ferroptosis induction in murine BMDMs *in vitro*. It was accompanied by the upregulation of Slc7a11 (a member of Xc^-^ system) and increased levels of ROS together with nuclear Nrf2 (**Figure 1**). Moreover, Slc7a11 knock-out was associated with higher susceptibility to iron-induced cell death, which was also shown to be independent of autophagy (83). The latter research described ferroptosis occurring in red pulp macrophages performing elevated erythrophagocytosis. This phenomenon was accompanied by increased ROS and LPO levels *in vivo* – the effects which were aborted by the Fer-1 use in vitro (84).

Since ferroptosis is not the only cell death modality that macrophages can undergo, and elevated ROS and LPO levels can arise in different scenarios, it is important to distinguish ferroptosis-related events from others. Wiernicki et al. revealed that in murine BMDMs membrane lipid peroxidation level was incomparably higher during ferroptosis than in other types of programmed cell death, with the prevailing fraction of phosphatidylethanolamine peroxide species (85). Furthermore, lipid ROS increased prior to cell permeabilization, unlike in non-canonical pyroptosis, and could be prevented by administrating Fer-1 (85).

Ferroptosis is considered one of the inflammatory cell death types (86), however, the knowledge on whether its occurrence in immune cells, macrophages in particular, affects the production of inflammatory mediators and downstream signaling pathways is still scarce. A study conducted by Xia et al. shed light on this topic as it demonstrated that murine MHV-A549-infected ferroptotic macrophages secreted IL-6, whereas the level of this cytokine significantly decreased after Fer-1 treatment (87). This data suggests that ferroptosis may enhance the inflammatory response of macrophages, however, further research is needed to validate the results.

### 2.4 The differential susceptibility of M1 and M2 macrophages to necroptosis and ferroptosis

The basal expression levels of cIAP family proteins, along with other components of cell death pathways, are closely associated with the variable susceptibility of macrophages to cell death during their differentiation process (88), and polarization state (89–91). The degradation of cIAPs prompted by the administration of SMAC mimetics results in either RIPK1-dependent apoptosis or necroptosis, when caspase-8 is concurrently inhibited (92, 93). M1-like murine and human macrophages treated with SM show increased susceptibility to necroptosis compared to their M2-like counterparts (89, 90). This vulnerability is attributed to classical inducers of the M1 subset, namely IFNγ and LPS treatment, which in BMDMs upregulate MLKL, Z-DNA/RNA binding protein 1 (ZBP1), and RIPK3 – the key molecules involved in necroptosis (89). In human macrophages, both type I and type II IFNs upregulate MLKL while downregulating miR324-5p, a direct regulator of MLKL expression, rendering them more susceptible to various necroptosis inducers (94). During influenza virus (IAV) infection, diminished miR324-5p expression in hMDMs contributes to controlling viral spread (94). MLKL expression is maintained at high levels by IFN signaling to ensure swift necroptosis in both BMDMs and hMDMs (**Figure 1**), leading to its classification as a constitutive IFN-regulated effector of necroptosis (CIREN) (94, 95). These pro-necroptotic effects of IFNs have implications in autoimmunity (95) and murine models of sterile sepsis induced by TNF (48). Furthermore, IFNβ suppresses IL-4-induced M2 polarization of BMDMs by controlling α-ketoglutarate/succinate ratio (96). However, there is also evidence that type I IFNs promote M2 polarization (97), and the resolution of bacterial inflammation by enhancing efferocytosis and reprogramming of macrophages to a pro-resolving phenotype (98).

Intensive macrophage cell death is also observed as a result of simultaneous caspase and TGF-activated kinase 1 (TAK1) inhibition, which prevents the phosphorylation of RIPK1 (46, 91, 99). Notably, this treatment selectively triggers necroptosis in M2 macrophages, while M1 macrophages, which rely on alternative survival signals (AURKA and GSK3β), are largely resistant (91).

In the case of ferroptosis, interesting results were provided by two different studies characterizing the capacity of both primary human and murine macrophages to handle iron. They revealed that M2-polarized macrophages displayed a higher rate of iron internalization and release when compared to M1 cells (100, 101). Additionally, Agoro et al. demonstrated that iron overload in vitro promoted M2 phenotype and decreased the LPS-induced pro-inflammatory response of M1 macrophages (102). These findings suggested that the activation state of macrophages determined their susceptibility to ferroptosis, which was later proved in mice (103, 104). Indeed, in a study performed by Kapralov et al. both inflammatory macrophages and microglia showed higher resistance to ferroptosis inducer RSL3 compared to non-polarized (M0) or M2 cells under both *in vitro* and *in vivo* conditions. Mechanistically, M1 cells had a higher content of inducible nitric oxide synthase and increased production of NO^•^, which could inhibit 15-lipoxygenase – an enzyme capable of producing lipid peroxides (**Figure 1**). Additionally, NO^•^ could also replace GPX4 in the cellular anti-ferroptotic machinery (103). Interestingly, 15-lipoxygenase plays a reverse function in the absence of ferroptosis inducer and is associated with the generation of pro-resolving lipid mediator RvD1 in satiated macrophages (105). In another study, Piattini et al. demonstrated that GPX4 loss in M2 cells led to their ferroptosis, while GPX4-deficient inflammatory macrophages maintained their functions and cell survival. Although NO^•^ supplementation alleviated ferroptosis in the M2 subset, iNOS inhibition in M1 cells could not induce lipid peroxidation. Thus, these results suggest that some other antioxidant mechanisms were responsible for controlling ROS levels in GPX4-deficient inflammatory macrophages (104). Noteworthy, the increased activity of heme oxygenase 1 (HO-1) might serve as another factor affecting M2 macrophage susceptibility to ferroptosis due to its potential to release Fe^2+^ from heme (**Figure 1**), as was implied by Li et al. (106). Contrarily, Fuhrmann et al. demonstrated that a hypoxic environment prevented hMDMs from RSL3-induced ferroptosis via decreasing nuclear receptor coactivator 4 (NCOA4)-dependent ferritinophagy and increasing mitochondrial ferritin level (107). Although the polarization state of macrophages was not assessed, hypoxia is known to promote the M2 phenotype (108). Therefore, it indicates that different M2-polarizing stimuli may have a distinct impact on the ferroptosis pathway, or that there is a varying connection between phenotype and susceptibility to ferroptosis in murine and human cells.

## 3 Cross-interactions among pyroptosis, necroptosis, and ferroptosis in macrophages

One of the earliest observations on pyroptosis-necroptosis crosstalk was that the release of the mature form of IL-1β can derive from ripoptosome and RIPK3-mediated NLRP3 activation (109, 110). Essential for this process seemed to be MLKL, which once activated was able to create pores in the cell membrane. This led to potassium efflux, thereby triggering the activation of inflammasome and IL-1β processing (**Figure 1**). Moreover, the subsequent IL-1β release seemed to be GSDMD-independent (111). Now, the evidence supporting these observations in macrophages is still growing (112–115). For example, Polykratis et al. have recently shown that RIPK3-MLKL-mediated necroptosis in macrophages was also accompanied by NLRP3 activation and IL-1β secretion (115). Interestingly, all these findings indicate the contribution of necroptosis signaling in pyroptosis. A reverse scenario has been recently described (116), in which disrupted mitochondrial homeostasis and mtROS enabled N-GSDMD to switch to a mitochondrial initiator of necroptosis (**Figure 1**). Moreover, Feng et al. challenged the classical model stating that cell death accompanying inflammasome activation should always be pyroptosis. They demonstrated that the bacteriolysis of *Staphylococcus aureus* USA300 strain in infected macrophages induces AIM2-mediated necroptosis (117).

At the crossroads of apoptosis, necroptosis, and pyroptosis, the pivotal regulator, caspase-8, orchestrates both necrotic cell death and the induction of pro-inflammatory gene expression, including NLRP3 (118–121). Upon activation of death receptors, TLR3, TLR4 as well as intracellular nucleic acid sensors RIG-I and ZBP1 (122, 123), caspase-8 is recruited to intracellular signaling complexes. Its functional role depends on the interplay with other partners, and even subtle alterations in the composition of caspase-8 complexes can give rise to contradicting physiological outcomes, such as the switching between macrophage death modalities or between cell death and cell activation (119). Notably, in the absence of an X‐linked inhibitor of apoptosis (XIAP), extrinsic caspase-8 promotes pyroptotic GSDMD processing, resulting in the demise of macrophages lacking both inflammasome and apoptosis signaling components (caspase-1, -3, -7, -11 and BID) (124). In addition to RIPK1, caspase-8 can cleave RIPK3 (125). Primed caspase-8-deficient macrophages exhibit elevated IL-1β production due to unrestricted activity of RIPK3 (126). Furthermore, caspase-8 can assemble an ASC-caspase-1 complex, culminating in caspase-1-dependent cell death and the release of IL-1β and IL-18 (119, 120).

Emerging evidence indicates that macrophages exhibit a flexible response to stimuli, leading to the induction of various cell death pathways (127). It has already been demonstrated that *Salmonella* Typhimurium infection in macrophages can induce pyroptosis (128) and necroptosis (129). Moreover, such evidence has recently been shown for human respiratory syncytial virus (RSV) (72). Bedient et al. revealed that macrophages infected with RSV activated NLRP3-ASC inflammasome, caspase-1, as well as RIPK3-MLKL. Intriguingly, ASC/NLRP3 deficiency in macrophages led to a substantial loss (72-75%) of lytic cell death, while inhibition of either caspase-1 or RIPK3 individually resulted in 46% and 54% loss, respectively (72). Considering the association of RIPK3-MLKL with NLRP3 activation, it appears that ASC-NLRP3 inflammasome contributes to both pyroptosis and necroptosis in RSV-infected macrophages. Furthermore, both types of lytic cell death may promote the amplification of lung inflammation during RSV infection (72).

Some supporting evidence showing that, indeed, the simultaneous induction of pyroptotic and necroptotic pathways in macrophages can drive inflammation has recently arisen in case of sepsis. Chen et al. demonstrated that mice with double depletion of RIPK3/GSDMD or MLKL/GSDMD exhibited higher protection against septic shock and multi-organ injury (130). A bone marrow transplantation from Ripk3-/-Gsdmd-/- to WT mice enhanced the protection against sepsis in such animals, suggesting that both myeloid and non-myeloid cells contributed to the disease progression. *In vitro* experiments from the same study revealed that macrophages lacking RIPK3 and GSDMD were resistant to both pyroptosis and necroptosis as well as cytokine production and release (130). All these findings suggest that pyroptotic macrophages together with necroptotic ones may contribute to inflammation and sepsis progression. However, it remains unclear whether these cell death pathways mutually influenced each other or if they were simply simultaneously activated without further crosstalk. Moreover, the possible occurrence of pyroptosis and necroptosis in other immune cells and their subsequent impact on sepsis progression cannot be excluded.

The concurrent occurrence of macrophage pyroptosis and necroptosis, along with its impact on exacerbating tissue damage in sepsis, can also be implied from the later studies by Russo et al. They focused on galactin-1, which increased levels can be found in sera from human patients with sepsis (131). The authors showed that galactin-1 is released by both pyroptotic and necroptotic macrophages, which is associated with LPS-induced lethality (131). Thus, targeting both lytic cell death types in macrophages may constitute a novel promising therapy to alleviate septic conditions. Interestingly, there are findings which show that Bcl-2, as well as necrosulfonamide (NSA), can attenuate both pyroptosis and necroptosis in macrophages (132, 133).

The relationship between ferroptosis and pyroptosis or necroptosis is not yet fully established. A study conducted by Kang et al. on the sepsis model demonstrated that GPX4 knock-out in macrophages, followed by increased lipid peroxidation, resulted in GSDMD cleavage mediated by both caspase-1 and caspase-11 (134). Since GPX4 is an antioxidant defense enzyme, its ablation could make macrophages more susceptible to ferroptosis and subsequent ROS generation could also activate inflammasome leading to pyroptosis. Thus, GPX4 knock-out can exacerbate the development of sepsis by possibly activating both cell death pathways. To further test this hypothesis, the expression levels of other ferroptosis markers (e.g. SLC7A11) as well as mitochondria morphology in GPX4-deficient macrophages should be assessed. Nuclear translocation of Nrf2, which is another regulator of ferroptosis, is downregulated in *Salmonella* Typhimurium-infected macrophages via IFN-1/RIPK3/PGAM5 axis (**Figure 1**). This leads to ROS-mediated mitochondrial dysfunction which can be a sign of cells undergoing ferroptosis (129). Interestingly, these events occur simultaneously with the induction of the classic necroptotic pathway, suggesting that both cell death modalities can be activated in infected macrophages. *Mycobacterium tuberculosis*, an intracellular pathogen known to cause macrophages to undergo necroptosis (135), can also prompt them to exhibit hallmarks of ferroptosis, that can be abolished by Fer-1 (136). While it is still not clear whether ferroptosis and necroptosis take place concurrently in the infected macrophages, ferroptosis might potentiate other cell death modalities to support the release of bacteria.

In this section, we explored the crosstalk between PCDs in macrophages. Interestingly, there is a clear involvement of pyro-, necro-, and ferroptosis in intracellular bacterial and viral infections as well as sepsis. In these settings, the initiation of multiple death programs seems to intensify the release of pathogens which can support disease progression. On the other hand, the inhibition of macrophage death can hamper these processes. Therefore, research into the molecules able to simultaneously inhibit lytic PCDs seems to be a worthwhile pursuit. At the same time, more extensive studies are required to discern the role of dying macrophages in diverse infectious conditions, beyond those mentioned above. In the following sections of this review, we will focus on discussing macrophage death within the context of more investigated illnesses – namely atherosclerosis and cancer.

## 4 Cell Death in atherosclerotic plaques: pyroptosis, necroptosis, and ferroptosis in macrophages

Atherosclerosis, the most prevalent form of cardiovascular disease, is primarily characterized by persistent low-grade inflammation as well as the gradual buildup of lipids in the intimal space of medium and large arteries (137). Among diverse cell types, macrophages affect the pathological progression of atherosclerosis, exerting both favorable and detrimental functions. They participate in the uptake of proatherogenic, oxidized low-density lipoproteins (oxLDLs), thus transforming into foam cells, which facilitate the development of atherosclerotic plaques (138, 139). In the early stages of the disease, this internalization may represent a beneficial adaptive mechanism of macrophages to manage lipid deposits. However, as the plaque advances, the regulation of cellular turnover becomes disrupted (140), and foam cells undergo both apoptotic and nonapoptotic forms of cell death. When inadequately cleared (141), these cells contribute to the formation of an acellular necrotic core (NC), fostering instability of advanced lesions. The destabilization of plaques is undesirable, as it poses the risk of atheroma rupture, bleeding, and thrombosis. For a long time, the necrotic debris within lesions was attributed solely to the passive lysis of uncleared apoptotic cells. However, research has revealed the involvement of various other forms of PCD in the formation of necrotic core and plaque destabilization (139, 142). Once developed NC advances along with the disease progression and its size correlates with the instability of the plaque (143). Thus, the occurrence of PCDs may influence disease outcomes. Here, we explore how three cellular death modalities in macrophages – pyroptosis, necroptosis, and ferroptosis – affect atherosclerosis progression. We discuss the occurrence of lytic PCDs in human plaques as well as possible triggers and modulators of these pathways in the atherosclerotic milieu. Finally, we analyze macrophage cell death in the context of its subpopulations observed in lesions.

### 4.1 Evidence for pyroptosis, necroptosis, and ferroptosis activation in macrophages within human atherosclerotic plaques

#### 4.1.1 Pyroptosis: a flame in atherosclerosis

Epidemiological studies reveal the presence of pyroptotic markers in human carotid atherosclerotic plaques (144–146) with NLRP3 emerging as a pivotal component implicated in atherosclerosis progression. Zheng et al. demonstrated high aortic expression of NLRP3 in patients with coronary atherosclerosis, which correlated with both disease severity and atherosclerotic risk factors such as smoking, hypertension, diabetes mellitus, and dyslipidemia (146). Subsequently, Shi et al. confirmed strong expression of NLRP3, ASC, caspase-1, IL-1β, and IL-18 in unstable atherosclerotic plaques, as opposed to stable ones (147). Notably, the NLRP3 inflammasomes were primarily localized in macrophages and foam cells (148) and associated with cholesterol crystals inside and outside these cells (147). The formation of cholesterol crystals is considered a significant factor contributing to atherosclerotic plaque expansion and rupture (149). They are abundantly present within foam cells and have already been shown to trigger pyroptosis in macrophages (150).

Similarly, activated caspase-1, together with IL-1β and IL-18 were identified in human advanced atherosclerotic plaques as opposed to non-atherosclerotic vessels and early-stage disease lesions. It was also demonstrated that cleaved caspase-1 co-localized with macrophages around the NC, while cleavage of apoptotic caspases was not observed (148, 151). Furthermore, Puylavert et al. showed that NT-GSDMD was localized in macrophage- and VSMC-rich regions of the plaques (152). However, this study did not include a comparison with plaque-free arteries, nor did it establish a correlation with the stage of the disease (152). Immunofluorescence and single-cell RNA sequencing analyses revealed that atherosclerotic macrophages localized in the NC of plaque exhibited higher expression of GSDME (145). This elevated expression was accompanied by increased levels of IL-1β and caspase-3 as compared to the non-necrotic areas within the same plaque (145).

#### 4.1.2 Necroptosis: unveiling molecular triggers

The necroptotic pathway is also known to be activated in human atherosclerotic plaques, especially in macrophages, and is associated with the formation of NCs. For instance, by a comprehensive gene expression analysis Karunakaran et al. identified elevated levels of *RIPK3* and *MLKL* mRNAs in human atheroma compared to disease-free control arteries (71). A notable increase was observed in symptomatic plaques in comparison to asymptomatic individuals (71). Moreover, MLKL phosphorylation was confirmed in clinical atherosclerotic samples, specifically in close proximity to the NC in advanced plaques, but not in early lesions (71). The upstream molecule, RIPK1, was highly expressed in human macrophages, whereas exhibited a low presence in vascular smooth muscle cells-rich regions in human carotid atheromas (153). Another study reported increased levels of the RIPK1-RIPK3 complex in atherosclerotic plaques with NCs (144). However, contradictory gene expression data indicated higher levels of RIPK1 in early atherosclerotic lesions than in regions with more severe plaques (154).

#### 4.1.3 Ferroptosis: connecting dots

The direct evidence of ferroptosis induction within human atherosclerotic plaques remains limited. The involvement of ferroptosis in atherosclerosis can be inferred indirectly, as indicated by the accumulation of ROS, lipid peroxidation in macrophages, atheroma hemorrhage, and iron deposition, all of which are notable features of advanced atherosclerotic plaque (139). However, a recent analysis of single-cell RNA sequencing data from human atheromas revealed that macrophages associated with symptomatic plaques upregulated *FTH1* and *FTL* genes encoding heavy and light chains of ferritin, respectively (155). These results align with the previous data on increased colocalization of iron, ferritin accumulation, and macrophage hemoglobin scavenger receptor (CD163) or hepcidin in advanced human plaques (156, 157). Collectively, these results indicate that there is a subset of macrophages in human atheromas that retain bound iron which should decrease their likelihood of undergoing ferroptosis. In this research topic, Sarad et al. reported a subtype-specific expression of core ferroptosis genes (e.g. *Cp, Hells, Slc40a1*) in inflammatory versus tissue-resident macrophages (158). This observation suggests a link between ferroptosis and inflammatory microenvironment appearing at a very early stage of atherogenesis. Their findings indicate that Nrf2 deficiency in aortic macrophages leads to subtype-specific transcriptomic changes associated with cell death pathways (158).

### 4.2 Triggers and modulators of macrophage pyroptosis, necroptosis, and ferroptosis within atherosclerotic milieu

A literature analysis revealed that the inflammatory forms of LDLs, particularly oxLDLs, serve as triggers for all three types of death in macrophages. They also modify cell susceptibility to other stimuli. These observations imply a nuanced interplay among pyroptotic, necroptotic, and ferroptotic pathways in the context of atherosclerosis progression. However, the majority of findings in this field originate from *in vitro* studies employing diverse macrophage types, including cell lines. Additionally, these studies often involve various forms of oxLDLs with imprecisely controlled compositions. Furthermore, such investigations frequently focus on the pathway associated with only one of the three cell death modalities. In the next section, we take a closer look at the role of oxLDLs along with other molecules, conditions, and processes implicated in the induction and regulation of macrophage death within the context of atherosclerosis. The existing knowledge is also organized and summarized in **Table 1**.

**Table 1:** Lytic PCDs in macrophages in the context of atherosclerosis: inducers and mechanisms.

Trigger	Concentration and exposure	Macrophage cell type/lines	Cell death type	Mechanism/molecular association	Inhibitor	Other cell death types investigated	Ref.
oxLDL	100 µg/mL 48hrs	Human MDMs	pyroptosis	CD36-mediated ROS generation and activation of NLRP3/caspase-1 pathway, DNA fragmentation, and caspase-1-dependent production of IL-1β, IL-18, IL-33, TNFα, IL-6	Ac-YVAD-CHO (caspase 1 inhibitor) and CD36 blocking antibody reduced caspase-1-dependent cytokine production and cell lysis	Not apoptosis	(151)
	50-200 µg/mL 1-24hrs			ROS-mediated activation of NLRP3 that leads to caspase-1-dependent IL-1β production; possible involvement of cathepsin B pathway (lysosomal rupture)	z-YVAD-fmk (caspase-1 inhibitor), cytohalasin D NAC (ROS scavenger), CA-074 Me (catepsin B inhibitor) reduced IL-1β production	–	(159)
	50 µg/mL 24hrs		ferroptosis	Increased levels of ROS, LDH, GSH and lipid accumulation associated with downregulation of GPX4 by lnc-MRGPRF-6:1	Knockdown of lnc-MRGPRF-6:1 alleviated the hallmarks of ferroptosis	–	(160)
	50-100 µg/mL 24hrs (ref. 161) 50 µg/mL 24hrs (ref.162)	THP-1-derived macrophages	pyroptosis	lncRNA overexpression increases *TXNIP* mRNA levels (ref.161) or HIF-1α-mediated induction of ROS signaling pathway (ref.162) which lead to NLRP3 inflammasome activation	lncRNA- and HIF-1α-specific siRNAs reduced the levels of inflammasome components as well as IL-1β and IL-18	–	(161) (162)
	50 µg/mL 24hrs		ferroptosis	Increased levels of ROS, LDH, GSH and lipid accumulation associated with downregulation of GPX4 by lnc-MRGPRF-6:1	Knockdown of lnc-MRGPRF-6:1 alleviated the hallmarks of ferroptosis	–	(160)
	100 µg/mL 48hrs			Increased level of iron and decreased levels of GPX4 and LC3 associated with upregulated expression of HIF-1α	PX478 (HIF-1α inhibitor) alleviated lipid accumulation and increased the levels of GPX4 and LC3.	–	(163)
	25-50 µg/mL 24hrs	Murine BMDMs	pyroptosis	CD36-TLR4-TLR6-mediated oxLDL recognition and CD36-mediated oxLDL uptake result in insoluble crystals formation, activation of NLRP3 inflammasome and IL-1β secretion	cytohalasin D, YVAD-fmk, ZVAD-fmk (pan-caspase inhibitor) inhibited IL-1β secretion	–	(164)
	100 µg/mL 24hrs		necroptosis	ROS dependent cell death associated with upregulated expression of RIPK3 and MLKL proteins	DPI (diphenyleneiodonium) reduced the levels of ROS and expression of RIPK3 and MLKL.	Caspase-1 independent cell death	(71)
	100 µg/mL 24hrs	Murine peritoneal macrophages	pyroptosis	Phosphorylated STAT3 promoted GSDME transcription associated with augmented caspase-3 activity and increased mRNA levels of TNFα, IL-1β, MCP-1, and IL-6	MCC950 (NLRP3 inhibitor) and siRNA-mediated knockdown of STAT3 reduced GSDME expression	GSDME serves as a switch from apoptosis to pyroptosis	(145)
	150 µg/mL + zVAD 12hrs		necroptosis	Cell death induced as a result of switch from apoptosis due to caspase-8 inhibition	Cell death is completely abolished in RIPK3-deficient macrophages	Treatment with oxLDL alone leads to cell apoptosis	(165)
	150 µg/mL 48hrs	RAW264.7 macrophages overexpressing RIPK3	necroptosis	Cell death associated with overexpression of RIPK3	–	–	(165)
	50 µg/mL 24hrs	RAW264.7	ferroptosis	Increased levels of Fe2+, lipid peroxides and downregulated levels of GPX4, FTH1 and SLC7A11 accompanied by overexpression of IDH1	siRNA-mediated knockdown of IDH1 alleviated hallmarks of ferroptosis and activated Nrf2 pathway	–	(166)
oxLDL + high glucose	100 µg/mL ox-LDL + 25 mmol/l D-Glu 24hrs	THP-1-derived macrophages	pyroptosis	NLRP3 inflammasome activation associated with oxidative stress, and ROS production	Spermine alleviated the expression of pyroptosis-associated NLRP3, N-GSDMD, IL-1β and IL-18 and reduced oxidative stress via inducing Nrf2 pathway	The involvement of Nrf2 suggest a potential link to ferroptosis	(167)
oxLDL + ferric amonium citrate (FAC)	50 µg/mL ox-LDL 24 hrs + 20 µM FAC 6hrs	THP-1-derived foam cells	ferroptosis	Increased level of ROS and HO-1 associated with reduced level of GPX4 and elevated expression of IL-1β and IL-18	Autophagy mediated SIRT1 activation inhibited foam cell ferroptosis caused by iron overload	The involvement of IL-1β and IL-18 suggests a potential link to pyroptosis	(168)
Serum starvation 24 – 48 hrs	Prior treatment with oxLDL 100 µg/mL 48 hrs	THP-1-derived foam cells	necroptosis	RIPK1/RIPK3 and MLKL-dependent cell death	5-aminolevulinic acid-mediated sonodynamic therapy disrupts necrosome formation and MLKL oligomerization and shifts the cells towards apoptosis	Apoptosis, necroptosis and necrosis occur simultaneously	(144)
Inflammation	LPS 50 ng/mL 16 hrs + IFN-γ 10 ng/mL 6 hrs	Murine BMDMs	necroptosis	HIF-1α enhances necroptosis of inflammatory macrophages by elevated mtROS production, decreased OXPHOS, and ATP depletion through upregulating miR-210 and downregulating miR-383	HIF-1α knockout in BMDMs prevented the LPS/IFNγ-induced decrease in ATP production	–	(169)
HUA + ox-LDL	15 mg/dL HUA 24hrs followed by 100 µg/mL ox-LDL 24hrs	THP-1 and RAW264.7-derived foam cells	ferroptosis	Decreased levels of NRF2, GPX4 and SLC7A11, aggravated mitochondrial damage and autophagy dysfunction	TBHQ (Nrf2 inducer) and rapamycin (autophagy inducer) reversed the effects of HUA treatment	–	(170)
Autophagy deficiency in foam cells	50 µg/mL oxLDL 48 hrs	THP-1-derived foam cells	ferroptosis	Nrf2 accumulation which leads to ROS generation and downregulation of GPX4 and xCT	ML385 (Nrf2 inhibitor) restored the levels of antioxidant mediators	–	(171)
Erythrophagocytosis	Jak2VF red blood cells at 10:1 ratio overnight	Murine BMDMs, Human MDMs	ferroptosis	Increased lipid peroxidation and decreased cellular viability in murine and human macrophages; the effects were observed in untreated (M0) or pretreated with IL-4 (M2) murine macrophages	Liproxstatin-1 alleviated the effects of erythrophagocytosis in both murine and human cells	Not pyroptosis, nor necroptosis	(172)
	Red blood cells at 80:1 ratio 0.5-4 hrs	Murine BMDMs		Elevated labile iron accumulation and lipid peroxidation associated with simultaneous increase in FTH1 and HO-1 levels	UAMC-3203 and UAMC-3206 (Fer-1 analogues) prevented RSL3 and FAC induced cell death	Not apoptosis nor necroptosis	(173)
Cigarette tar	175 µg/mL 12hrs	THP-1-derived macrophages	ferroptosis	Elevated lipid peroxidation and reduced levels of FPN, SLC7A11 and GPX4, increased expression of hepcidin mediated by NF-κB pathway	BAY 11-7082 (NF-κB inhibitor) abrogated tar-induced upregulation of hepcidin and increased expression of antioxidant mediators	Partially could be pyroptosis not necroptosis	(174)

#### 4.2.1 OxLDL-induced pyroptosis in macrophages

It has been demonstrated that in hMDMs, oxLDL induced the expression of NLRP3, caspase-1, and ASC, leading to caspase-1 activation, macrophage lysis, and the release of IL-1β and IL-18 (151, 159). It was later proven that oxLDL-induced pyroptosis in hMDMs relied on CD36 and was associated with robust ROS generation and NLRP3/caspase-1 pathway activation. Noteworthy, although apoptotic caspases (caspase-3, -6, -8, and -9) were also activated in oxLDL-treated cells, only the use of caspase-1 inhibitor led to a significant suppression of cell lysis (151). In contrast, Nogieć et al. found neither the release of IL-1β nor an increased accumulation of its precursor form in non-stimulated human monocyte-derived foam cells (hMDFCs) treated with oxLDL (175). This discrepancy is likely due to a time- and/or dose-dependent issue, as the treatment involved a relatively low concentration of oxLDLs for an extended incubation period, which should mimic *in vivo* conditions more accurately. Moreover, these cells tended to follow a necrotic pathway distinct from pyroptosis and did so more promptly than short-term-induced foam cells (175). Noteworthy, oxLDL is a known inducer of miR-155 expression (176, 177), which correlates with atherosclerosis (178). Given that hMDFCs, obtained after prolonged exposure to oxidized LDL, respond to inflammasome activation preferentially with pyroptosis combined with other types of necrotic death (175), it is plausible to think that miR-155 effector mechanism lies in switching between cell death pathways, regulating the immunogenic cell death burden.

Oxidized LDL may contribute to pyroptosis in macrophages also through the upregulation of the expression of GSDME (145). Interestingly, GSDME augments caspase-3 activity, which shifts the mode of macrophage cell death from apoptosis to pyroptosis, thereby promoting the progression of atherosclerosis. Moreover, GSDME deficiency significantly decreases serum levels of inflammatory factors, such as IL-1β, TNFα, and monocyte chemoattractant protein-1 (MCP-1), resulting in the suppression of both inflammation and disease development (145).

Recently, it was described that long non-coding RNAs (lncRNAs) can be involved in the regulation of oxLDL-induced pyroptosis (161, 162). Indeed, both linc00657 (161) and AC078850.1 (162) potentiated pyroptosis in THP-1-derived macrophages. Interestingly, the latter molecule acted (161) in conjunction with hypoxia-inducible factor 1-alpha (HIF-1α) to promote NLRP3-mediated pyroptosis and foam cell formation in atherosclerosis cases (162).

As mentioned earlier, CD36 has been documented to play a role in oxLDL-mediated macrophage pyroptosis (151). CD36 is a scavenger receptor that cooperates with the TLRs heterodimer TLR4-TLR6 in the recognition of oxLDLs as well as mediates its uptake from the surrounding environment. It appears to function as a dual signal in NLRP3 inflammasome-mediated macrophage pyroptosis activation since macrophages from transgenic mice with CD36 knock-out exhibited reduced levels of *Il1a*, *Il1b* and *Nlrp3* mRNA, as well as lower IL-1β serum level. Furthermore, such animals were protected from atherosclerosis (164).

#### 4.2.2 Hyperglycemia-induced pyroptosis in macrophages

Qiu et al. generated interesting results related to pyroptotic pathway inhibition for atherosclerosis treatment (167). They focused on spermine, a natural cellular metabolite that exerted protective properties against macrophage pyroptosis induced by hyperglycemia and oxLDL via activation of Nrf2 signaling pathway and the inhibition of NLRP3 inflammasome. Furthermore, spermine significantly reduced the generation of ROS, and release of IL-1β and LDH (167). Nie et al. found that under diabetic conditions, the expression of GSDMD and caspase-1 in macrophages was increased, and the recognition of cytoplasmic double-stranded DNA by AIM2 inflammasome might play an important role in initiating pyroptosis in macrophages under hyperglycemia (179). While these studies may not have focused directly on atherosclerosis, they are still worth mentioning, since diabetes promotes pro-inflammatory gene expression in macrophages and contributes to atherosclerotic lesions development (137, 180).

#### 4.2.3 OxLDL-induced necroptosis in macrophages: beyond caspase inhibition

Caspase inhibition in peritoneal murine macrophages was shown to switch oxLDL-induced apoptosis to necroptosis, with complete abolition of necrotic cell death in RIPK3-deficient murine peritoneal macrophages (165). Furthermore, oxLDLs alone could induce cell death in the RAW264.7 macrophage cell line overexpressing RIPK3. Additionally, *in vivo* studies revealed increased RIPK3 levels in atherosclerotic macrophages during lesion development and the degree of necroptosis was found to correlate with the level of RIPK3 deletion in tested animals (165). Importantly, Karunakaran et al. reported that in BMDMs, atherogenic forms of LDL increased both RIPK3 and MLKL expression through direct activation of their promoters, confirming that oxLDLs could induce necroptotic cell death even without synthetic caspase inhibitors (71). Overall, these findings suggest that oxLDLs can independently lead macrophages to undergo necroptosis when the expression of RIPK3 in atherosclerotic plaques reaches a certain threshold. Interestingly, the induction of necroptosis by oxLDLs was independent of inflammasome activation, as cells deficient in caspase-1 or treated with caspase-1 inhibitors still underwent necroptotic cell death in response to oxLDL. Moreover, they did so to the same extent as wild-type or untreated cells (71).

#### 4.2.4 Impact of hypoxia, nutrient deficiency, and HDL intervention on macrophage necroptosis

Hypoxic and nutrient-deficient conditions in atherosclerotic plaque can contribute to its growth, instability, and eventual rupture. For instance, serum starvation could induce necrotic types of cell death in THP-1-derived foam cells. Although apoptosis, necroptosis, and necrosis were observed under insufficient nutrient supply, results following pre-treatment with necrostatin-1 suggested that nearly half of the deceased cells were attributed to necroptosis (144). Furthermore, researchers demonstrated a significant increase in RIPK1 and RIPK3 protein levels, RIPK1-RIPK3 complexes, and MLKL oligomerization (144). The interplay of hypoxia, macrophages, and atherosclerosis was assessed by Karshovska et al. (169). They showed that the additional deletion of HIF-1α in myeloid cells from ApoE-deficient mice reduced atherosclerosis and NC formation by limiting macrophage necroptosis. *In vitro* experiments on inflammatory HIF-1α-deficient BMDMs from the same study confirmed increased oxidative phosphorylation and ATP levels, alongside reduced ROS production and necroptosis (169).

Recently, it has been elucidated that anti-atherogenic high-density lipoproteins (HDLs) may attenuate NC development in atherosclerotic plaques. Kluck et al. demonstrated that the HDL treatment of both THP-1 and murine peritoneal macrophages suppressed necroptosis induced by TNF or oxLDL in the presence of the pan-caspases inhibitor. This protective effect was contingent on the HDL receptor SR-B1 and activation of Akt kinase (181).

#### 4.2.5 OxLDL-induced ferroptosis in macrophages

A growing body of evidence suggests that oxLDLs can also trigger ferroptosis in macrophages. Li et al. demonstrated that oxLDL treatment in RAW264.7 macrophages increased Fe^2+^ levels, induced lipid peroxidation, and downregulated the protein levels of GPX4, FTH1, and SLC7A11 (166). Foam cells were also characterized by overexpression of isocitrate dehydrogenase (IDH1), whereas the inhibition of IDH1 activated Nrf2 pathway, thereby reducing macrophage ferroptosis and foam cell development (166). Interestingly, a high level of uric acid (HUA) appeared to contribute to the oxLDL-induced foam cell formation from THP-1 and RAW264.7 cells in a similar manner as IDH1 (170). Namely, HUA inhibited Nrf2 signaling pathway and promoted ferroptosis. It also impaired mitochondrial function and autophagy in macrophage-derived foam cells. These effects could be restored by ferroptosis inhibitor Fer-1 (170).

#### 4.2.6 Regulating ferroptosis in macrophages in the context of atherosclerosis

Significantly, IL-37 can mitigate ferroptosis-associated oxidative stress and Nrf2 inhibition. IL-37 enhances cellular viability and promotes the nuclear translocation of Nrf2 in THP-1 macrophages treated with oxLDLs (182). Long non-coding RNA lnc-MRGPRF-6:1 is another type of molecule that can contribute to foam cell ferroptosis. It appears that lnc-MRGPRF-6:1 may promote ferroptosis in THP-1 and hMDMs by suppressing GPX4 (160). Nevertheless, it remains to be elucidated whether this molecule only inhibits *GPX4* mRNA or if it also downregulates the expression of Nrf2 located upstream.

Nrf2 is typically regarded as a master regulator of cellular antioxidant defense (183). However, recent findings by Peng et al. have shown that in the late stages of atherosclerosis, autophagy deficiency can lead to Nrf2 accumulation in THP-1 foam cells, resulting in the generation of ROS and the induction of ferroptosis (171). These data suggest that, in specific circumstances, Nrf2 may negatively influence overall cell survival and promote disease progression.

HIF-1α is a well-known transcription factor recognized as an essential mediator of oxygen homeostasis (184). HIF-1α is upregulated in human atherosclerotic macrophages and its inhibition alleviates lipid accumulation in oxLDL-induced THP-1-derived macrophages *in vitro* by suppressing ferroptosis and activating autophagy (163). It would be of interest to assess whether the Nrf2/HIF-1α ratio in macrophages changes as a result of ferroptosis occurrence and plaque formation, and if a higher proportion could serve as a prognostic factor for patients.

Remarkably, ferroptosis is not limited to foam cells. It also occurs in M0 and M2 macrophages following the erythrophagocytosis of Jak2^VF^ red blood cells, as demonstrated by Liu et al. (172). The Jak2^VF^ mutation is found in human patients with atherosclerosis, whereas in murine models is linked to accelerated disease due to increased ferroptosis of macrophages (172). Cigarette tar is another factor that promotes the development of atherosclerotic lesions and is associated with the upregulation of hepcidin in plaque macrophages. *In vitro* studies on THP-1 have shown that tar induced ferroptosis via NF-κB-activated hepcidin/FPN/SLC7A11 pathway. It has been further observed that disrupting this signaling either pharmacologically or genetically can alleviate the progression of atherosclerosis *in vivo* (174).

### 4.3 Pyroptosis, necroptosis, and ferroptosis in the context of macrophage subsets in atherosclerosis

The incontrovertible involvement of macrophages in atherosclerosis development is acknowledged. However, the temporal changes that heterogenic populations of macrophages undergo functionally within the dynamic and complex milieu of atherosclerotic plaques are less recognized (185). The spatiotemporal distribution, phenotypic diversity, and death of macrophages within lesions assume a crucial role in shaping the microenvironment and influencing the fate of atheromas. As the macrophage heterogeneity in atherosclerotic plaque has been extensively reviewed elsewhere (186), we aim to discuss them in the context of necrotic PCDs occurring within the atherosclerotic microenvironment.

#### 4.3.1 M1 and M2 polarization and cell fate in atherosclerosis

M1 macrophages typically dominate in the rupture-prone shoulder regions of the plaque and adjacent to the NC (187). The buildup of lipids not only leads to foam cell formation but also fosters M1-like polarization of the cells and establishes a mutually reinforcing relationship with them, creating a self-sustaining loop. Much like M1 macrophages, foam cells are commonly situated in close proximity to the NC. In contrast, M2 macrophages, recognized for their anti-inflammatory and repair functions, primarily reside in more stable regions of plaques, near newly formed blood vessels, and in areas of ongoing repair (187). Hence, managing the balance between pro-inflammatory M1 and anti-inflammatory M2 macrophages through repolarization or targeted depletion may offer significant therapeutic advantages.

Although direct evidence is currently lacking, it is plausible that M1 macrophages may demonstrate heightened sensitivity to pyroptosis. Specifically, the activation of the inflammasomes and the subsequent release of pro-inflammatory cytokines, which are distinctive features of pyroptosis, might align with the functional pro-inflammatory characteristics of M1 macrophages.

The data obtained by Hao et al. and Ali et al. implied that under conditions of inflammation and stress, M1 macrophages may be predisposed toward necroptosis (89, 90). Given the persistent inflammatory milieu characteristic of atherosclerosis, it is conceivable that cells undergoing necroptosis manifest a pro-inflammatory phenotype. This hypothesis was substantiated to some extent in a study conducted by Karshovska et al., wherein the heightened susceptibility of inflammatory macrophages to necroptosis was demonstrated to be dependent on HIF-1α-mediated metabolic reprogramming toward an inflammatory state (169).

Although the differential susceptibility of macrophages to ferroptosis is not yet elucidated in the context of atherosclerosis, the general viewpoint is that murine M1 macrophages, in comparison with M0 and M2, exhibit increased resistance to pharmacologically-induced ferroptosis (103). This resistance seems to hinge on elevated NO production and the secure sequestration of iron through ferritin within M1 macrophages (100). On the other hand, alternatively activated macrophages appear to be more prone to ferroptosis in the absence of GPX4, indicating a diminished antioxidant capacity in these cells (104). Moreover, iron overload, while seemingly ineffectual on macrophages, leads to ferroptosis upon their exposure to oxLDLs (168). The demise of M2 cells from the atherosclerotic plaque could potentially worsen disease progression by downregulating the processes associated with these macrophages such as secretion of anti-inflammatory cytokines, efferocytosis of cellular debris, or tissue repair (188).

The lack of data on macrophage polarization in the context of cellular death in atherosclerotic milieu renders it challenging to anticipate which subpopulations of macrophages are sensitive to either of the lytic PCDs and how it could be leveraged for therapeutic purposes. This intricacy is further compounded by the ability of oxidized phospholipids to induce phenotypic switching in both murine M1 and M2 macrophages, giving rise to the so-called Mox phenotype (189). The existence of macrophages associated with high levels of iron adds another level of complexity to this issue.

#### 4.3.2 Mox and hemoglobin-associated phenotypes: antioxidant defense, potential ferroptosis resistance, and iron homeostasis

Mox macrophages, described by Kadl et al., were approximated to constitute one-third of total macrophages present in mouse atherosclerotic plaques (189). Mox phenotype is distinguished by Nrf2-mediated upregulation of the expression of antioxidant enzymes, including HO-1, thioredoxin reductase 1, and sulfiredoxin-1 (189). Although their resilience against ferroptosis remains unexplored, it is plausible that the Mox subset may confer increased resistance to this particular form of PCD. Interestingly, *in vitro* experiments involving the exposure of macrophages to oxidized phospholipids resulted in elevated levels of HO-1, ferritin, and hepcidin (190), suggesting that a microenvironment rich in oxLDL could promote iron retention in macrophages, preventing them from undergoing ferroptosis.

Oxidative stress within macrophages can be induced by various compounds present in atherosclerotic plaques such as hemoglobin found at sites of intraplaque hemorrhage. Boyle et al. identified a hemorrhage-associated subpopulation of macrophages, termed Mhem, characterized by high hemoglobin content (191). Correspondingly, a macrophage subset named M(Hb) was identified for its ability to engulf hemoglobin-haptoglobin complexes (192). Both Mhem and M(Hb) macrophages are predominantly located around human hematomas and are notably absent in stable and hemorrhage-free plaques. They exhibit elevated expression levels of CD163 and Nrf2-dependent HO-1 (191, 192). The characteristic iron retention by these subsets can be attributed to TLR-mediated signaling that induces hepcidin expression, an effect similar to one achieved by LPS stimulation (193, 194). Due to the upregulation of cholesterol efflux proteins and resistance to foam cell transformation, Mhem and M(Hb) macrophages are considered atheroprotective. Additionally, as a result of increased expression of ferroportin, they have low intracellular levels of iron and ROS (192). These features can be a sign of adaptation to the atherosclerotic microenvironment and may protect them against cell death. However, they can contribute to elevated iron levels in the plaque, the subsequent accumulation of which in other macrophage populations activates pro-inflammatory M1 phenotype and promotes the demise of cells possibly via ferroptosis (100). Interestingly, Mhem and M(Hb) murine counterparts have not been described yet. However, recent studies on murine models have shown that erythrophagocytosis by macrophages induces non-canonical ferroptosis that manifests by increased HO-1 expression and contributes to plaque destabilization (173).

### 4.4 Section summary

Here, we have discussed the involvement of three modes of macrophage death in atherosclerosis development and progression. Noteworthy, recent single-cell studies in human atherosclerotic plaques confirmed the presence of two inflammatory subsets characterized by the expression of inflammasome components, IL-1β, and TNF (195). Additionally, a macrophage cluster with unique upregulation of genes responsible for iron storage and metabolism was identified (155), further linking the atherosclerotic environment to specific types of cell death. However, there is currently a lack of spatiotemporal analysis regarding the respective contributions of intraplaque pyroptosis, necroptosis, and ferroptosis to atherogenesis. Neverthless, targeting lytic PCDs within the macrophages may hold promise for alleviating the disease. In this regard, the efficacy has recently been demonstrated for baicalin – a natural flavonoid isolated from medicinal herbs (68, 196), and dimethyl fumarate – an anti-inflammatory agent approved for the treatment of relapsing-remitting multiple sclerosis (69, 197). However, further studies are necessary to validate whether the results obtained *in vitro* or in murine models can be translated to the human body. Moreover, extensive research is needed to determine which signaling pathway should be inhibited at various stages of atherosclerotic plaque and NC development. An alternative approach might involve shifting macrophages undergoing regulated necrotic cell death toward regulated non-necrotic death modalities, e.g. apoptosis and autophagy. Another avenue involves enhancing the clearance of necrotic cells through the modulation of macrophage metabolism, potentially utilizing agents such as resolvins (198). Nonetheless, there is a long road ahead before any of these concepts can be developed into therapies. Even then, targeting macrophage death to resolve inflammatory microenvironment should come along with dietary changes and a reduction in the intake of LDLs, which while oxidized aggravate atherosclerosis and serve as potent inducers of macrophage necrotic PCDs.

## 5 Tumor microenvironment: pyroptosis, necroptosis, and ferroptosis in macrophages

### 5.1 The impact of macrophages and pro-inflammatory cell death on tumor microenvironment

Macrophages constitute a highly prevalent type of leukocytes within the tumor microenvironment (TME) that are able to survive in regions with low oxygen levels, known as hypoxic areas (199). Such tumor-associated macrophages (TAMs) are also regarded to follow the paradigm of M1- and M2-like phenotypes (200, 201). In general, due to their pro-inflammatory characteristics, M1-like macrophages are typically identified as tumor-suppressing and associated with extended overall patient survival (200). For instance, they can accomplish this by recruiting CD8+ T and NK cells as well as secreting cytokines, which further enhance anti-cancer immune response (201). On the contrary, anti-inflammatory M2-like TAMs contribute to tumor development by promoting angiogenesis as well as the production of factors that stimulate cancer cell proliferation and survival (202). These macrophages are more frequently found in TME than M1-like type and correlate with poor prognosis (200).

The elimination of tumor-supporting TAMs constitutes a potential approach for cancer therapies, as there is much evidence indicating their role in promoting tumor growth (203). In this context, the induction of a pro-inflammatory type of macrophage death seems to be particularly appealing. It not only eliminates cells with often pro-tumorigenic functions but also leads to the release of molecules that support an anti-tumor immune response. Indeed, DAMPs and cytokines produced during immunogenic PCDs were documented to trigger antigen-presenting cell maturation and/or activation (204), antigen-specific CD8+ T cell response (205) as well as chemotaxis and cytotoxicity of NK cells (206). Thus far, numerous studies have concentrated on the induction of pyroptosis, necroptosis, and ferroptosis specifically in tumor cells for potential cancer therapy, as discussed elsewhere (207–210). In contrast, the amount of research focusing on these PCDs in macrophages within TME is significantly lower. Moreover, their impact on tumorigenesis seems to vary under different conditions, thus being intricate. Here, we gather factors triggering macrophage pyroptosis, necroptosis, and ferroptosis in TME. We also compare the mechanisms of these processes, their links to TAMs polarization as well as the overall effects on tumor progression and organize these data in **Table 2**. Our approach aims to provide a comprehensive overview and summarize current knowledge in this field.

**Table 2:** Lytic PCDs in macrophages in the context of cancer: mechanisms and outcomes.

Tumor type	Cell death pathway	Macrophage cell type/lines	Inducer	Mechanism/molecular association	Link to macrophage polarization	Association with disease outcome	Ref.
Head and neck squamous cell carcinoma	pyroptotic	Murine BMDMs, Human MDMs	Efferocytosis of apoptotic tumor cells	NLRP3 inflammasome activation and IL-1β release independent of GSDMD	–	Tumor growth progression	(211)
Breast cancer	pyroptotic	Murine TAMs and BMDMs, Human MDMs	S1PR1 signaling	NLRP3 inflammasome activation and IL-1β release which induced the production of VEGF-C in lymphatic endothelial cells	–	Pathological tumor lymphangiogenesis	(212)
	ferroptotic	Murine BMDMs, Murine TAMs	MIL88B/RSL3 nanocombination	Downregulated expression of GPX4 and elevated lipid peroxidation	Nanocombination triggered a switch from OXPHOS to glycolysis and the elevated expression of surface markers CD80 and CD86 and augmented production of IL-6, IL-12, and IL-1, marking a repolarization towards M1 type	Slowed down tumor growth	(213)
Colorectal carcinoma	pyroptotic	Murine BMDMs	dsDNA released from tumor cells post irinotecan (CPT-11) treatment	AIM2 inflammasome activation and IL-1β and IL-18 release	–	No association with disease outcome; AIM2 inflammasome activation correlates with irinotecan-induced intestinal injury	(214)
Leukemia	pyroptotic	Human MDMs, Murine peritoneal macrophages	DAMPs (e.g. ATP) released by pyroptotic cancer cells treated with CAR-T therapy	NLRP3 inflammasome activation, caspase-1 and GSDMD cleavage with subsequent IL-1β and IL-6 release	–	IL-1β is responsible for causing cytokine release syndrome which impedes the potential of CAR-T therapy	(215)
Glioma	pyroptotic	Human MDMs	Potentially LPS/bacteria and oxidative stress	Caspase-1/caspase-4/GSDMD axis activation	–	Tumor progression	(216)
Hepatocellular carcinoma Hepatocellular carcinoma	pyroptotic	Murine TAMs and BMDMs, Human MDMs	Sorafenib	Caspase-1-dependent release of IL-1β and IL-18, which mediate NK cells activation and subsequent tumor cell killing	Overall macrophage infiltration in tumors decreased while the proportions of M1 and M2 cells remained the same. Prior macrophage polarization had no impact on their pyroptosis	Tumor growth inhibition that is abolished by macrophage depletion	(217)
	necroptotic	Human TAMs, Murine TAMs and BMDM, Murine peritoneal macrophages	–	RIPK3 deficiency in TAMs resulted in decreased ROS production and caspase-1 activation which led to increased PPAR signaling and metabolic shift towards fatty acid oxidation	RIPK3 deficient cells exhibited M2-like phenotype manifested by elevated expression of Arg1 and CD206	Reduced level of RIPK3 in TAMs is associated with pro-tumorigenic phenotype which can be reversed by decitabine treatment	(218)
	ferroptotic	THP-1-derived macrophages, Murine TAMs	APOC1 inhibition	Elevated cellular iron and ROS content and reduced expression of GPX4, Nrf2, and SLC711A	APOC1 inhibition resulted in a shift towards M1 phenotype manifested by increased levels of CD86 and decreased levels of CD206, CD163, and Arg1	Reduced tumor progression, increased sensitivity to anti-PD-L1 therapy in mouse model	(219)
		Murine TAMs	xCT knock-out	Elevated levels of iron and MDA and decreased level of GSH	xCT is involved in macrophage polarization towards M2 phenotype via SOCS3-STAT6-PPARγ axis, which is reversed by xCT knock-out	Reduced tumor progression, increased sensitivity to anti-PD-L1 therapy in mouse model	(220)
Pancreatic ductal adenocarcinoma	necroptotic	Murine TAMs, Murine BMDMs Human TAMs	–	RIPK1 signaling prevents STAT1-mediated TAMs activation	RIPK1 inhibitor GSK’547 induced a shift of macrophages towards immunogenic phenotype manifested by upregulation of MHC-II, CD86, CD80, TNFα, and IFNγ	RIPK1-expressing murine TAMs supported tumor growth while inhibition of RIPK1 activity in TAMs promoted tumor suppression, and increased sensitivity to PD-1 and ICOS-based immunotherapies in mouse model	(221)
Cervical cancer	necroptotic	U937-derived macrophages	–	Tumor cells inhibited expression and phosphorylation of MLKL and necroptosis in macrophages	Macrophages cocultured with tumor cells released lower levels of IL-6 and TNF-α suggesting a shift in polarization from M1	–	(222)
Lung carcinoma	ferroptotic	Murine BMDMs, Murine TAMs	Dihydroartemisinin (DHA)	Decreased level of GPX4 and increased ROS and LPO levels which triggered DNA damage and subsequent DNA damage response and NF-κB signaling	Macrophages treated with (DHA) exhibited higher expression of CD86, IL-6, IL-12 and IL-1β	Potent tumoricidal activity	(223)
Nasopharyngeal carcinoma	ferroptotic	THP-1-derived macrophages	–	Exosomal MIF inhibited ferroptosis by restoring GPX4 production and reducing ROS level	Inhibition of MIF activity with ISO-1 reduced expression of Arg1 and CD163 upon stimulation	Reduced ferroptosis in macrophages was associated with increased lung metastasis in mice	(224)

TAMs stand for tumor-associated macrophages present in human tumor samples or in murine tumor models.

### 5.2 Macrophage pyroptosis in TME

#### 5.2.1 Factors inducing pyroptosis in TAMs

Looking for factors that trigger TAMs pyroptosis and have an impact on the TME and immune response, seems to be reasonable. Some studies demonstrate that either cancer cells or tumor-derived molecules can act as DAMPs causing inflammasome activation and pyroptosis in macrophages (211, 212, 214, 215, 225). Interestingly, it was also reported that pyroptosis and/or inflammasome activation in TAMs can arise as a side effect of various anticancer therapies (214, 215, 225, 226) or may be triggered by factors other than DAMPs (216, 227). In this section, we take a closer look at all this evidence.

##### 5.2.1.1 Tumor cells and their DAMPs

Cancer cells can act as factors activating pyroptotic molecules in macrophages. Lang et al. demonstrated that efferocytosis of apoptotic cancer cells by TAMs both *in vitro* and *in vivo* led to NLRP3 inflammasome activation, caspase-1 processing, and IL-1β secretion in those macrophages (211). This phenomenon resulted in immunosuppression and the promotion of tumor growth. Interestingly, in GSDMD KO mice a decreased tumor progression was not observed, which indicated the lack of a tumor-supportive role of GSDMD in models used in this study (211). Noteworthy, this research also included bulk RNA sequencing of CD11b+ myeloid cells sorted from human head & neck squamous cell carcinoma (HNSCC) tumors. Such analysis demonstrated an increase in *NLRP3* and *IL1B* gene expression in tumor-infiltrating myeloid cells, which was consistent with transcriptomic results from *in vitro* experiments. Moreover, tumor-derived CD11b+ cells were characterized by upregulated expression of efferocytosis-associated genes. These findings support the authors’ hypothesis that efferocytosis-induced NLRP3 inflammasome activation in tumor-associated macrophages may promote the pro-tumorigenic effects within TME.

The contrary impact of pyroptotic macrophages on tumor growth was proposed by Wang et al. (228). They suggested that inhibition of sphingosine kinase 1 (SPHK1) activity in TME cells along with induction of pyroptosis in TAMs might contribute to the suppression of tumor development. The concept was based on the findings that sphingosine, the substrate for SPHK1, can derive from cancer cells and act as a DAMP. Its accumulation can lead to pyroptosis in tumor-associated macrophages and further induction of anti-tumor immune response (228). While no publication delves deeper into and supports their hypothesis, there is a study focusing on related molecules. Weichand et al. showed that the SPHK1 product, which is sphingosine-1-phosphate, and its receptor play a prominent role in tumor progression (212). The authors demonstrated that S1P receptor 1 (S1PR1) signaling in TAMs promoted lymphangiogenesis via NLRP3-dependent IL-1β secretion. It was suggested that IL-1β, released from macrophages after NLRP3 activation, evoked the production of VEGF-C in lymphatic endothelial cells (212). Interestingly, S1PR1 knock-out in TAMs strongly reduced IL-1β level and lymphangiogenesis, though it did not affect tumor development in methylcholanthrene (MCA)-induced fibrosarcoma murine model (212). The above results show that there is a strong need for further research into sphingosine and its phosphorylated form in the context of TME, as well as other cancer-derived molecules potentially influencing macrophage pyroptosis.

##### 5.2.1.2 DAMPs released as a side effect of anticancer therapy

Inflammasome activation in TAMs was reported to be a possible outcome of anticancer treatment as many antitumor therapies can lead to the release of tumor-derived DAMPs (214, 215, 225, 226). For example, HCT-116 cells treated with irinotecan (CPT-11) secreted nuclear double-stranded DNA in exosomes that were subsequently engulfed by macrophages leading to the activation of AIM2 inflammasome and IL-1β release (214). Although the inhibition of AIM2 alleviated CPT-11-induced intestinal toxicity, it did not influence the drug’s anticancer efficacy (214). This data suggests that inflammasome activation in macrophages did not contribute to the antitumor immune response, but it triggered unwanted effects of CPT-11 treatment instead. It remains unclear whether IL-1β production was associated with macrophage pyroptosis.

Chemotherapy is not the only example of antitumor therapy, in which macrophage inflammasome activation seems to be a side effect. Recent findings indicate that chimeric antigen receptor (CAR) T cell therapy may result in extensive pyroptosis in B leukemic and other target cells (215). This treatment is accompanied by the release of large amounts of DAMPs e.g. ATP that can be picked up by macrophages and can further activate NLRP3 inflammasome leading to caspase-1 and GSDMD cleavage, as well as IL-1β maturation and secretion. Such processes, however, result in cytokine release syndrome (CRS) and CAR-T-related toxicities rather than effective antitumor immune response and cancer regression (215). This topic has been further explored by Deng et al., who proposed HMGB1 as one of the crucial DAMPs responsible for the strong side effects of this therapy (225). Their concept is derived from the fact that patients treated with CAR-T cells are characterized by high levels of HMGB1 (225).

Radiotherapy is another example of anticancer treatment that has an established contribution to the induction of macrophage pyroptosis. Liu et al. demonstrated radiation-induced NLRP3 inflammasome activation, IL-1β production, and pyroptosis in BDMDs in a dose-dependent manner. Moreover, they showed that macrophages were tolerant to relatively low doses of radiation. The mentioned effects were downregulated by knockout of NLRP3 both *in vitro *and *in vivo* (226). These observations provoke an interesting question, whether radiation-induced macrophage pyroptosis is favorable, and may stimulate an anticancer immune response, or mediate severe tissue damage instead. Nonetheless, it has not been further discussed in the literature.

##### 5.2.1.3 Factors other than DAMPs

Since macrophage pyroptosis can be induced by a range of factors, DAMPs may not be the exclusive initiators of this process in TME. A recent study on the correlation between the occurrence of TAMs pyroptosis and cancer progression utilizing single-cell RNA-sequencing analysis demonstrated that human glioma-infiltrating macrophages, MDMs in particular, had significant co-expression of *caspase-1* and *GSDMD* genes (216). Moreover, MDMs from high-grade gliomas showed increased transcripts of pyroptotic genes and their elevated infiltration was a characteristic of the high-risk group. Authors proposed LPS/bacteria and oxidative stress as potential initiators of MDMs pyroptosis since the signaling pathways responding to such factors were activated during macrophages’ progression toward the pyroptosis fate (216). It is particularly intriguing, considering evidence of the presence of bacteria and LPS in human gliomas (229). Since these components have already been shown to trigger macrophage pyroptosis, we hypothesize that bacterial LPS found in gliomas may induce pyroptosis in infiltrating MDMs. Its subsequent influence on the tumor microenvironment can promote cancer progression and lead to shorter overall patient survival. On the other hand, the observed oxidative stress signaling may be increased due to hypoxia, a common feature of the tumor microenvironment (230). Hypoxia has been recently proposed as a second signal to induce pyroptosis in LPS-primed macrophages leading to NLRP3 inflammasome activation, pro-caspase-1 and GSDMD processing, as well as IL-1β and LDH release (227). Thus, both studies together shed new light on the role of bacteria along with oxidative stress in inducing macrophage pyroptosis and promoting tumor progression.

#### 5.2.2 Advantageous induction of macrophage pyroptosis in anticancer therapy

Targeting TAMs pyroptosis may diminish the number of often pro-tumorigenic macrophages and promote antitumor immunity simultaneously. Several studies have suggested that the induction of macrophage pyroptosis can stimulate an anticancer immune response, thereby being favorable (217, 228, 231). Okondo et al. demonstrated that Val-boroPro, a nonselective inhibitor of post-proline cleaving serine proteases and a potential anticancer agent, triggered pyroptosis in monocytes and macrophages (231). Such a phenomenon may be vital for the high efficacy of Val-boroPro against cancers, as it was shown that the depletion of phagocytic cells significantly reduced Val-boroPro’s antitumor activity (232).

Another study by Hage et al. showed that macrophage pyroptosis is essential for the antitumoral effects of sorafenib, a multitarget kinase inhibitor approved for the treatment of hepatocellular carcinoma (HCC) (217). The efficacy of sorafenib depended on the release of interleukins 1β and 18 from pyroptotic macrophages, as well as subsequent induction of natural killer cell-mediated cytotoxicity against HCC (217).

### 5.3 Macrophage necroptosis in TME

Necroptosis in cancer cells was reported to be a tumor-suppressive mechanism, particularly serving as an alternative cell death pathway in apoptosis-resistant cells (233). However, there is still a lack of consistent and comprehensive data addressing the role of necroptosis mediators in the function of tumor-infiltrating macrophages. It appears that the outcomes of targeting necroptosis in TAMs vary depending on the specific protein that is inhibited. Moreover, the potential benefit from this approach is more complex when considering the link between necroptotic molecules and M1- and M2-like macrophage polarization.

#### 5.3.1 Outcomes of targeting different necroptotic molecules in TAMs

##### 5.3.1.1 RIPK1 inhibition in TAMs leads to M1-like polarization and tumor suppression

An elevated expression of RIPK1 was observed in both malignant epithelial cells and tumor-associated macrophages within human pancreatic ductal adenocarcinoma (PDA) (221). It was established that the pivotal factor for PDA progression was the presence of RIPK1 in the extra-tumoral compartment. Subsequently, it was elucidated that the selective inhibition of RIPK1 using GSK’547 induced a repolarization of TAMs toward an immunogenic phenotype, that led to the activation of adaptive immune responses and tumor suppression. Notably, such an outcome was substantiated through experiments in mice and organotypic models of human PDA. The inhibition of RIPK1 enhanced the effectiveness of PD-1 and ICOS-based immunotherapies in PDA-bearing mice (221). In the context of this review, it is worth noting that the tolerogenic phenotype exhibited by tumor-associated macrophages in PDA was reliant on RIPK1 but independent of RIPK3 and necroptosis (221).

##### 5.3.1.2 RIPK3 deficiency in TAMs leads to M2-like phenotype and tumorigenesis

In contrast to the role of RIPK1 as a central regulator of immune tolerance in PDA, the decreased expression of RIPK3 in macrophages associated with HCC correlated with tumorigenesis as observed in both murine models and human samples. Such a reduction in RIPK3 levels was also associated with the accumulation and polarization of TAMs toward an M2-like phenotype (218). Furthermore, in this study macrophages were subjected to treatment with decitabine, a hypomethylating drug evaluated in a clinical trial for liver metastasis and colorectal cancer therapy. As a result, the expression of RIPK3 in such macrophages was augmented which effectively reversed the immunosuppressive activities of TAMs within the HCC tumor microenvironment (218).

##### 5.3.1.3 Decreased MLKL leads to the reduction in both necroptosis and pro-inflammatory mediators

A high level of MLKL in peripheral blood mononuclear cells from cervical cancer patients was associated with improved overall survival (222). Notably, when the U937 macrophages were cocultured with cervical cancer cells, the decreased expression and phosphorylation of MLKL were observed along with reduced necroptosis. This was accompanied by the downregulation of pro-inflammatory mediators, suggesting a shift from M1 polarization and the potential establishment of a more immunosuppressive environment. Importantly, the overexpression of RIPK3 in macrophages reversed the impact of cancer cells on macrophage polarization and necroptosis (222).

#### 5.3.2 M1- vs M2-like macrophages susceptibility to necroptosis in TME

M1 and M2 macrophages display differential sensitivity to necroptosis-inducing stimuli, such as SMAC mimetics or TAK1 kinase inhibitors. These agents hold significant potential in the field of cancer therapy since cancer cells acquire resistance to apoptosis. Nevertheless, it is necessary to consider that necroptosis of tumor cells within the TME can result in TAMs-mediated immunosuppression (234, 235). Moreover, elevated susceptibility of M1 macrophages to the pro-necroptotic effects of SMAC mimetics may pose additional challenges for therapies based on them (89, 90). On the contrary, the alteration in the M1/M2 macrophage ratio, which stems from the increased susceptibility of M2 populations to necroptosis induction by TAK1 kinase inhibitors, provides a rationale for triggering necroptosis in anticancer therapy (91).

### 5.4 Macrophage ferroptosis in TME

So far, a large body of literature showed that ferroptosis activation in TME is linked to the depletion of M2 TAMs (236), their repolarization toward M1 type *in vitro* (213, 219, 223) as well as decreased tumor progression and metastasis (213, 219, 220, 223, 224). While no direct evidence of macrophage reprogramming *in vivo* has been obtained yet, it still seems that inducing ferroptosis in tumor microenvironment and/or tumor-associated macrophages may be advantageous.

#### 5.4.1 Ferroptosis induction reduces the number of M2-like macrophages and leads to M1-like phenotype repolarization

The evidence that ferroptosis-related genes affect the polarization state of macrophages has been previously provided by Hu et al. Their bioinformatical analysis of gene expression in HNSCC identified – suppressor of cytokine signaling 1 (SOCS1) and ferroptosis inhibitor FTH1 as prognostic factors. In this study, among tumor-infiltrating immune cells the former gene was associated with M1 macrophages, and the expression of the latter was mostly correlated with M2 type (237). Moreover, *FTH1* mRNA was elevated in lymph node metastasis. The authors further implicated that induction of ferroptosis may serve as an anticancer strategy capable of improving the efficacy of available immunotherapies in HNSCC. The experiments performed by Zhao et al. on a murine HNSCC xenograft model proved that ferroptosis inducer RSL3 successfully initiated this form of PCD in the grafts and also significantly reduced the number of M2 TAMs in the microenvironment (236). Although the number of M1 TAMs was not assessed, it is possible that RSL3 treatment led to macrophage repolarization. The supporting evidence provided by Gu et al. (213) showed that MIL88B nanoparticles containing RSL3 elicited ferroptotic stress in M2-polarized macrophages manifested by inhibition of GPX4 expression and high lipid peroxidation levels. Such a nanocombination also triggered metabolic reprogramming by switching from oxidative phosphorylation to glycolysis and induced M1 repolarization from M2 cells *in vitro*. Moreover, *in vivo* experiments showed an increase in the percentage of tumoricidal and metastasis-suppressing M1 cells in the breast tumor model (213).

The induction of macrophage ferroptosis constitutes a promising approach for resolving immunosuppressive microenvironment in HCC. Hao et al. discovered that APOC1 protein was overexpressed in HCC tissues and TAMs, while inhibition of *APOC1* gene shifted macrophage phenotype toward M1 (219). It was achieved by the induction of the ferroptotic pathway evidenced by increased iron and ROS content in the cells as well as decreased expression of GPX4, Nrf2, and SLC7A11. APOC1 knock-out in TAMs was shown to reduce proliferation, migration, and invasion of tumor cells. Moreover, it boosted sensitivity to anti-PD-1 therapy (219). Similarly, xCT-specific knock-down in murine model of HCC also inhibited M2-type polarization in TAMs via activating ferroptosis pathway (220). x-CT mediated macrophage ferroptosis additionally enhanced PD-L1 expression in these cells, thus improving the efficacy of antitumor treatment. Erastin-containing nanoparticles constructed by the researchers were effective in inducing macrophage ferroptosis and combined with anti-PD-L1 performed better than either of the monotherapies (220).

Lastly, dihydroartemisinin (DHA) is an antimalarial drug that was additionally proved to shift the polarization status of M2 macrophages toward M1 type both *in vitro* and *in vivo* in murine lung carcinoma model (223). As previously, repolarization was linked to macrophage ferroptosis manifested by increased levels of ROS and LPO. This, in turn, led to DNA damage and the associated cellular response which in turn activated NF-κB signaling pathway (223).

#### 5.4.2 Cancer-related molecules inhibiting TAMs ferroptosis

Noteworthy, cancer cells may target ferroptosis in macrophages to abolish its anticancer effects as was demonstrated previously (224). In this study exosomal macrophage migration inhibitory factor derived from CNE-2 tumor cells inhibited macrophage ferroptosis in lung metastasis, thus promoting disease spreading (224). In addition, an analysis of single-cell sequencing and transcriptome data obtained from melanoma and HNSCC tumors (238) indicated that CD86hi (M2-like) TAMs displayed an increased level of ferroptosis (238). Noteworthy, these cells were considered major contributors to immune therapy resistance (173). This implies that in some cases cancer cells may harness macrophage ferroptosis to promote disease development; hence, further studies are necessary to untangle this relationship.

### 5.5 Section summary

The data discussed above indicates that targeting macrophage pyroptosis for the preferred induction of antitumor immunity rather than driving the unwanted vicious circle of inflammation and toxicity can pose a challenge. Although the occurrence of TAMs pyroptosis within the tumor microenvironment has been extensively documented, it is still hard to reach a clear conclusion. The impact of macrophage pyroptosis on tumor development seems to depend not only on its inducer but also on the cancer type. The whole scenario can become even more intricate, considering the fact that certain tumors, e.g. gastric and colorectal cancer, are inflammation-driven (239). A more evident conclusion can be drawn for necroptosis. Based on the limited dataset, it seems that within TME the principal regulators of necroptosis primarily influence macrophage phenotype in a manner independent of cell death. Nevertheless, these findings suggest a potential therapeutic strategy that involves manipulating necroptosis components to target the polarization and metabolism of TAMs. So far, studies on macrophage ferroptosis in TME suggest that its induction may resolve the immunosuppressive microenvironment, promote M1-like polarization, and reduce tumor growth. Since it may improve the effectiveness of cancer immunotherapies, it should be an attractive candidate for further research.

## 6 Concluding remarks

Given the unique role of macrophages, the demise of these cells acquired exceptional significance (240–244). Despite the distinct molecular pathways through which pyroptosis, necroptosis, and ferroptosis are executed, the data summarized in this review unequivocally highlight remarkable flexibility and crosstalk among these mechanisms. It becomes evident that diverse modes of cell death can be initiated in response to the same stimulus, as exemplified by the uptake of oxLDLs by macrophages. It is still an open question whether the observed phenomenon represents the cumulative effect of various cell death modalities taking place in the mixture of cells or if it involves a mixed-type response within an individual cell, akin to the concept of PANoptosis (245). Differential susceptibility to death pathways induced by oxLDLs can also be caused by macrophage polarization states, which govern cell functions and phenotype. However, various subsets of plaque-associated macrophages described only in humans or in mice limit the ability to compare the processes occurring in the atherosclerotic milieu of the two species. Nevertheless, the occurrence of either lytic PCD aggravates the disease, as opposed to cancer, where the impact of macrophage death on tumor growth is contextually variable and depends on both the type of tumor and macrophage PCD modality. Thus, the research into the mechanisms governing macrophage death in atherosclerosis, e.g. various non-coding RNAs, could open new horizons for treating cancers in which targeting macrophages is of interest. Similarly, looking into the negative regulators of macrophage death in tumors could create new avenues for alleviating atherosclerosis.

The main limitation of the studies discussed in the review lies in the utilization of a dichotomic *in vitro* model of macrophage polarization, which dramatically simplifies the heterogeneity of TAMs or macrophages in atherosclerotic plaques observed in patients (76, 155, 195, 246). A further validation of macrophage activation subsets and their origin in humans is required to assess the susceptibility of tumor- and atherosclerosis-associated macrophages to various PCD modes. Nonetheless, it is conceivable that with time these discoveries, coupled with advanced techniques providing detailed insights into the spatial organization of tumors and atherosclerotic plaques at the single-cell level, will facilitate the development of innovative therapeutic approaches.

## Author contributions

MM: Visualization, Writing – original draft, Writing - review & editing. MS: Visualization, Writing – original draft, Writing - review & editing. MB: Conceptualization, Funding acquisition, Supervision, Writing – original draft, Writing – review & editing.
